# Supplementary figures and images for: Emerging Insights into Keratin 16 Expression during Metastatic Progression of Breast Cancer
Source: Cancers (Basel). 2021 Jul 31;13(15):3869. doi: 10.3390/cancers13153869 (PMC8345379; doi:10.3390/cancers13153869)

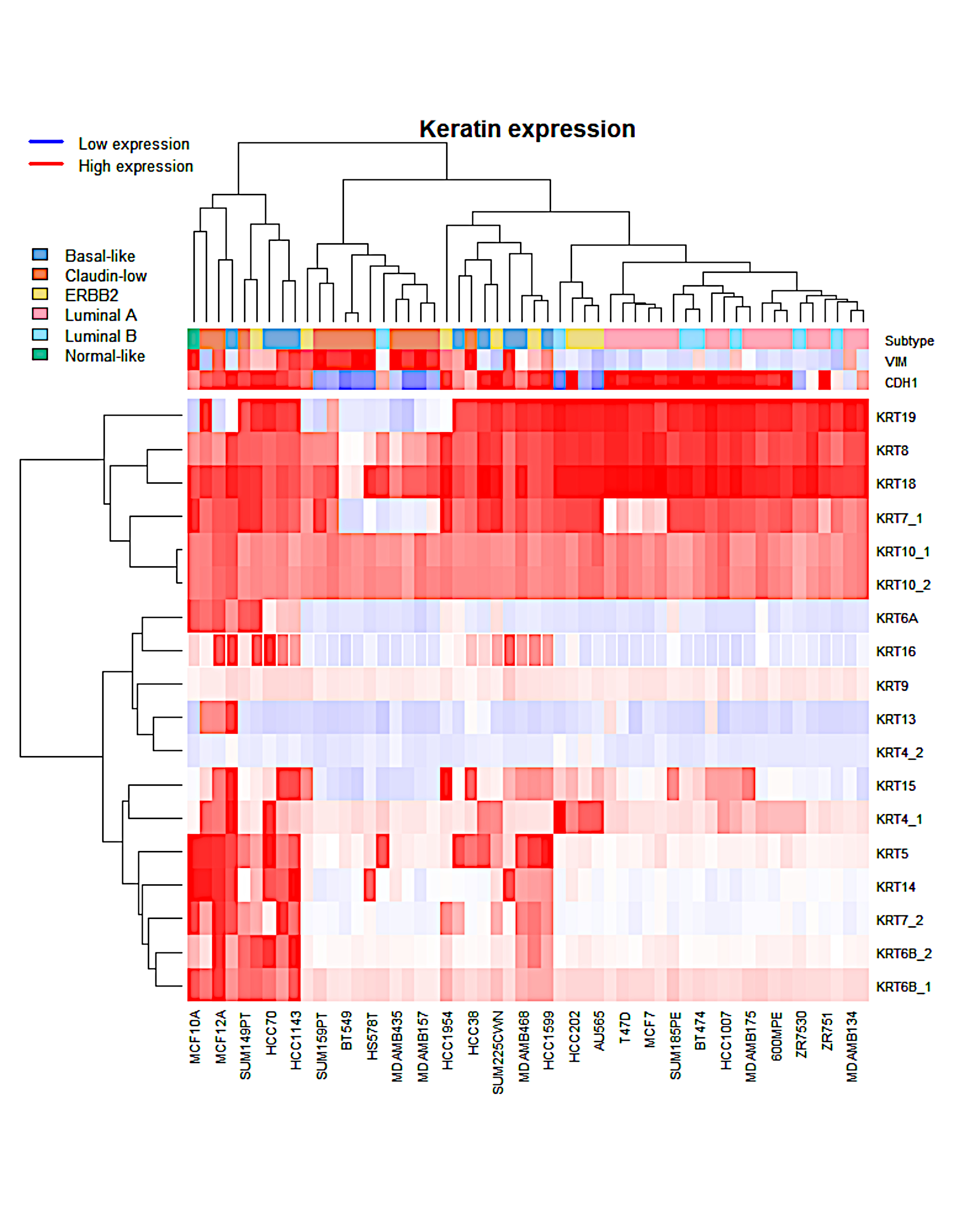

Supplement: Supplementary file 1 [file cancers-13-03869-s001.zip › cancers-1312672-supplmentary files/Figure S1.jpg]

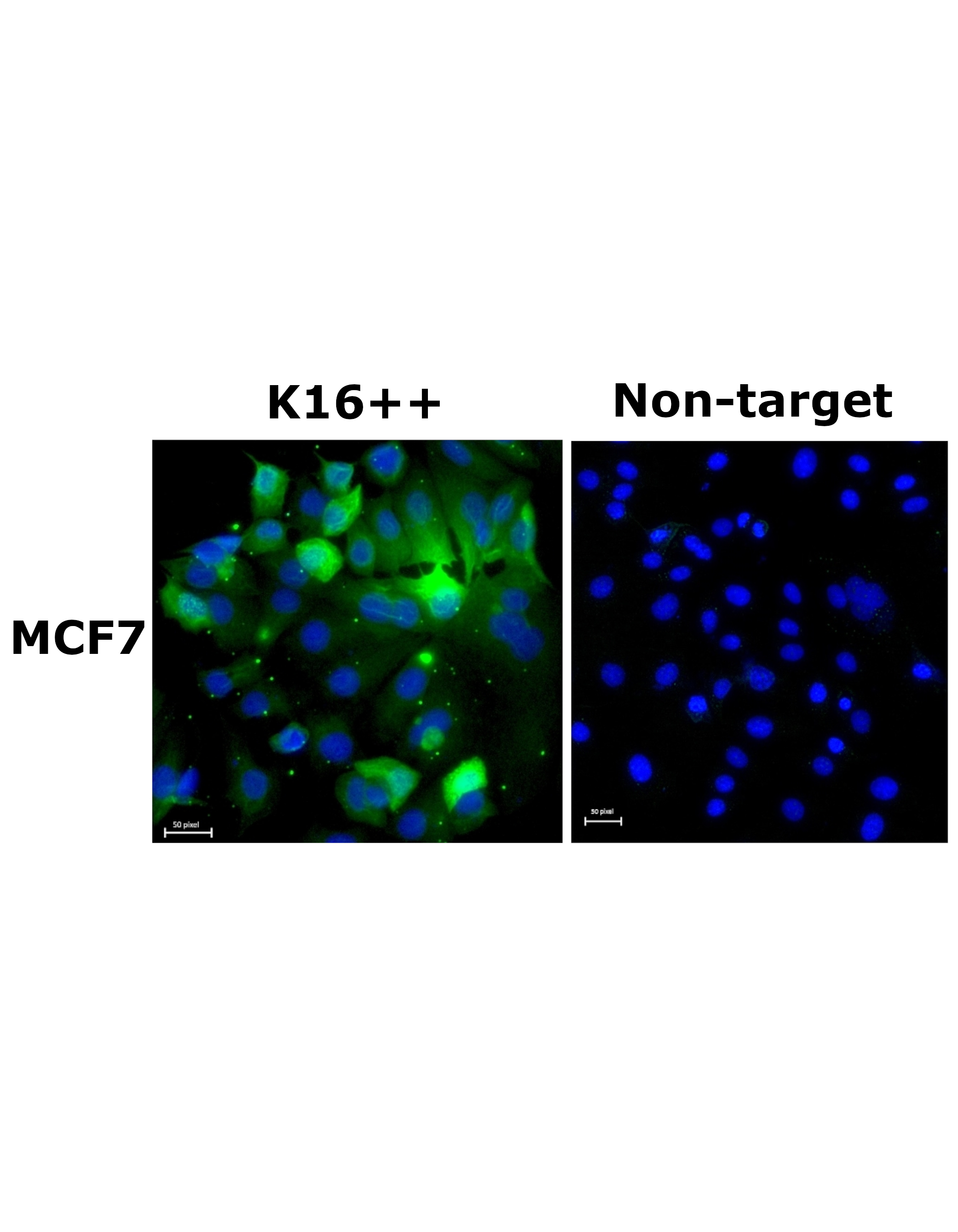

Supplement: Supplementary file 1 [file cancers-13-03869-s001.zip › cancers-1312672-supplmentary files/Figure S2.jpg]

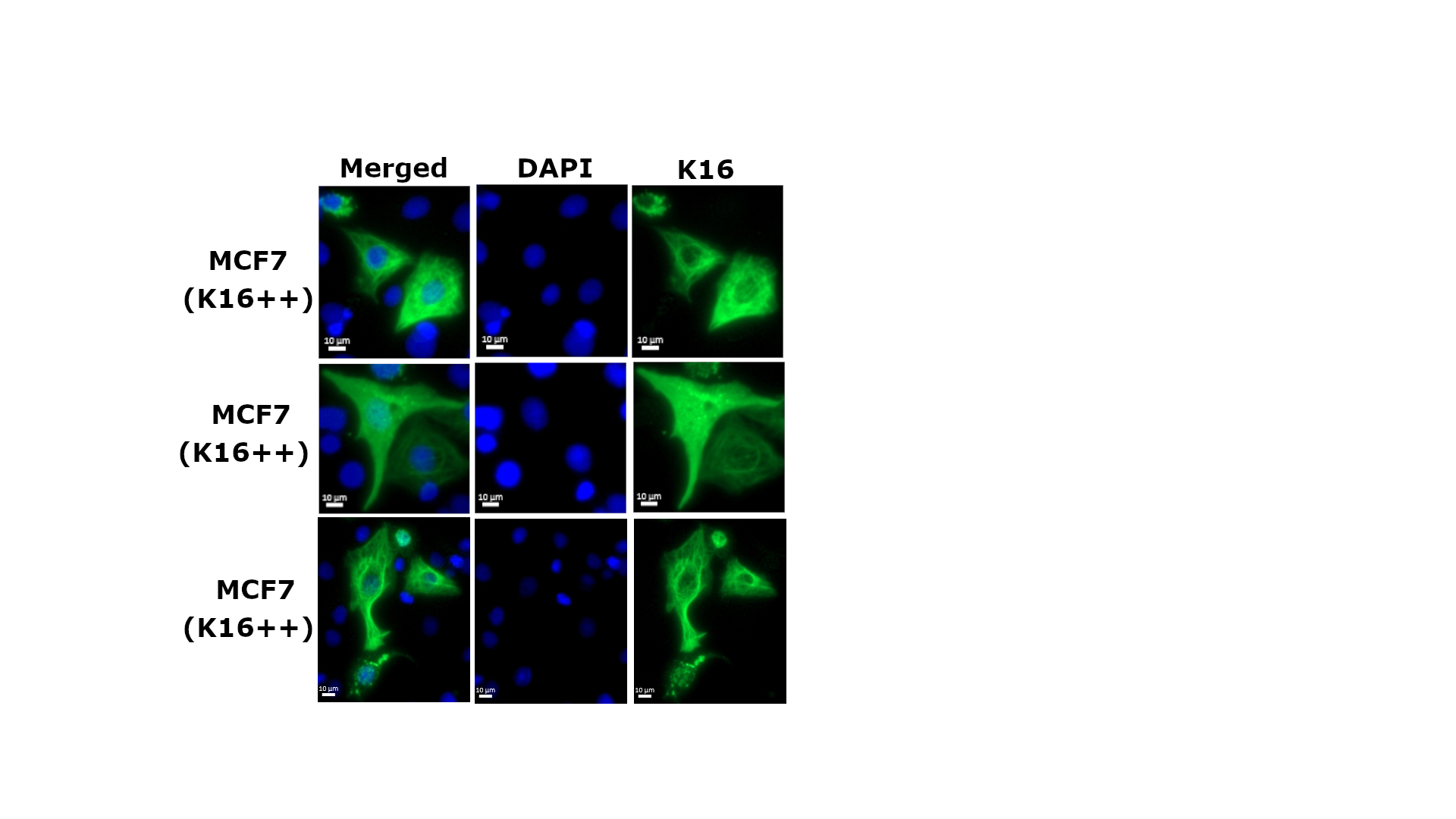

Supplement: Supplementary file 1 [file cancers-13-03869-s001.zip › cancers-1312672-supplmentary files/Figure S3.jpg]

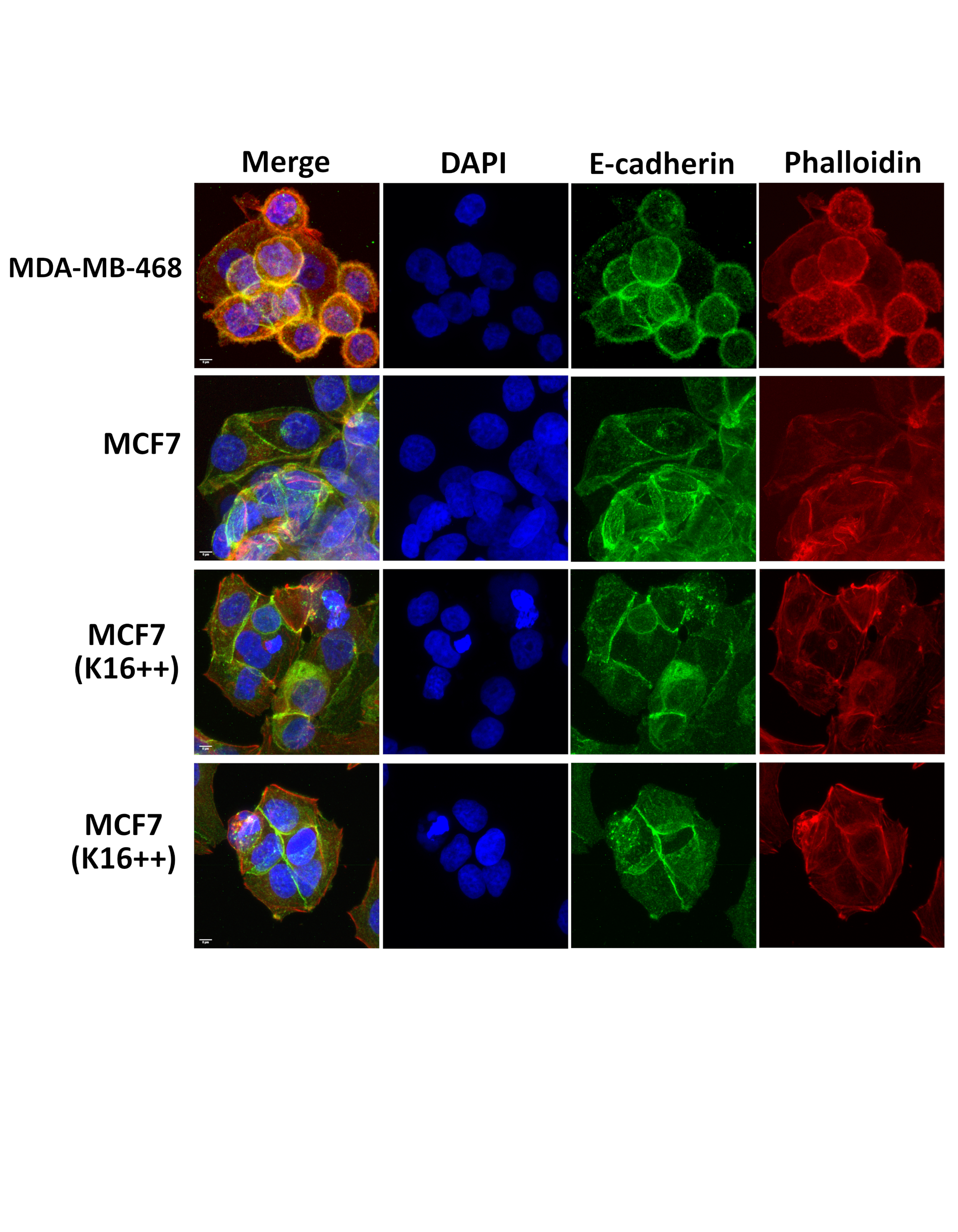

Supplement: Supplementary file 1 [file cancers-13-03869-s001.zip › cancers-1312672-supplmentary files/Figure S4.jpg]

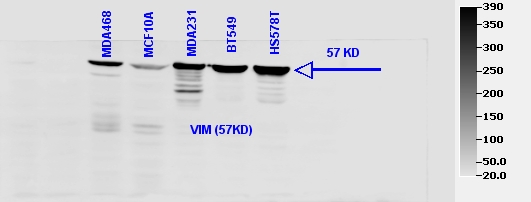

Supplement: Supplementary file 1 [file cancers-13-03869-s001.zip › cancers-1312672-supplmentary files/Original Images for Blots/Breast cancer cell lines _Basal Like_ VIM.jpg]

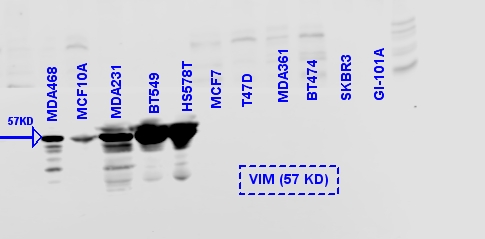

Supplement: Supplementary file 1 [file cancers-13-03869-s001.zip › cancers-1312672-supplmentary files/Original Images for Blots/Breast cancer cell lines _ Basal Like and Luminal_ VIM.jpg]

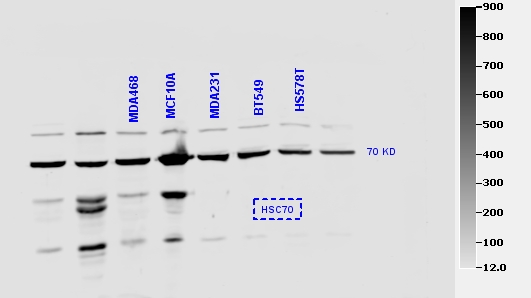

Supplement: Supplementary file 1 [file cancers-13-03869-s001.zip › cancers-1312672-supplmentary files/Original Images for Blots/Breast cancer cell lines _ Basal Like_HSC70.jpg]

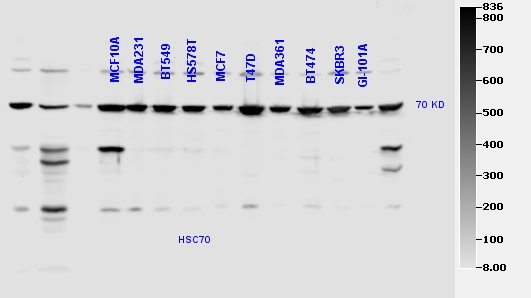

Supplement: Supplementary file 1 [file cancers-13-03869-s001.zip › cancers-1312672-supplmentary files/Original Images for Blots/Breast cancer cell lines _ Luminal_HSC70.jpg]

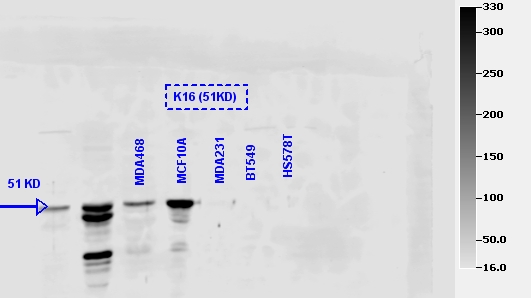

Supplement: Supplementary file 1 [file cancers-13-03869-s001.zip › cancers-1312672-supplmentary files/Original Images for Blots/Breast cancer cell lines _Basal Like_K16.jpg]

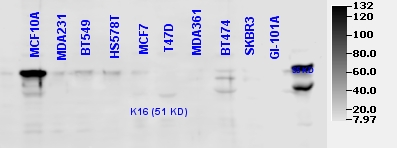

Supplement: Supplementary file 1 [file cancers-13-03869-s001.zip › cancers-1312672-supplmentary files/Original Images for Blots/Breast cancer cell lines _Luminal_ K16.jpg]

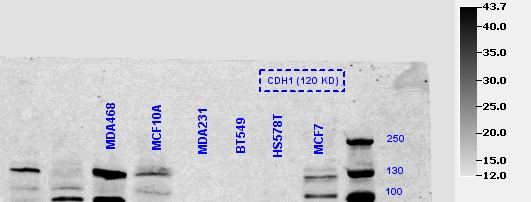

Supplement: Supplementary file 1 [file cancers-13-03869-s001.zip › cancers-1312672-supplmentary files/Original Images for Blots/Breast cancer cell lines_ Basal Like _CDH1.jpg]

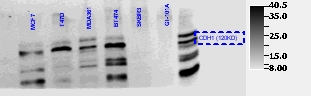

Supplement: Supplementary file 1 [file cancers-13-03869-s001.zip › cancers-1312672-supplmentary files/Original Images for Blots/Breast cancer cell lines_Luminal_CDH1.jpg]

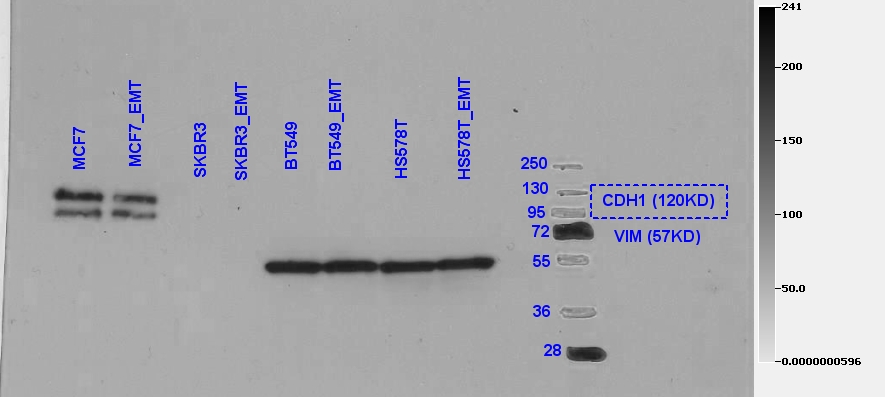

Supplement: Supplementary file 1 [file cancers-13-03869-s001.zip › cancers-1312672-supplmentary files/Original Images for Blots/EMT_CDH1and VIM.jpg]

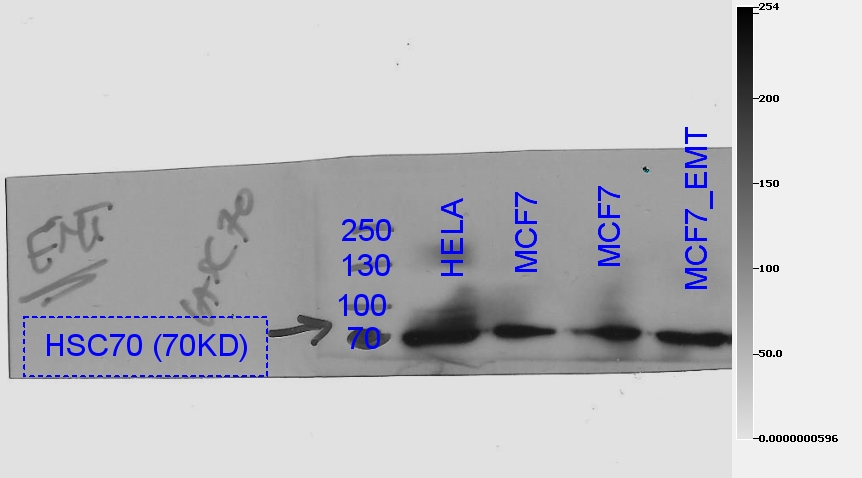

Supplement: Supplementary file 1 [file cancers-13-03869-s001.zip › cancers-1312672-supplmentary files/Original Images for Blots/EMT_HSC70.jpg]

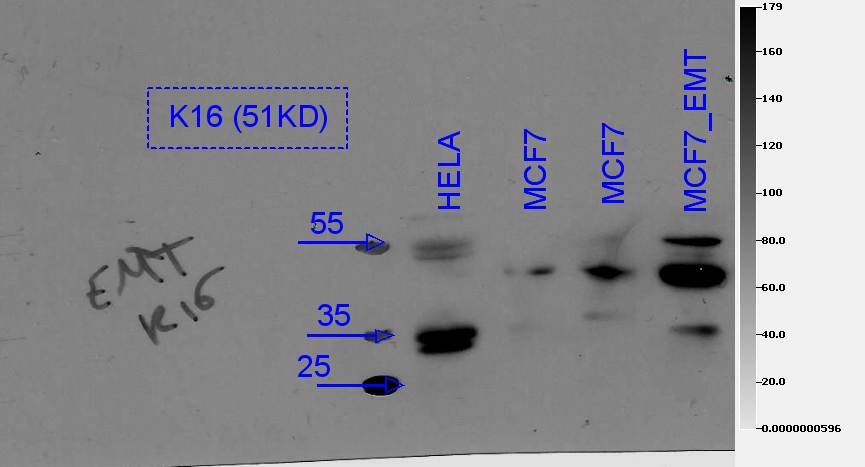

Supplement: Supplementary file 1 [file cancers-13-03869-s001.zip › cancers-1312672-supplmentary files/Original Images for Blots/EMT_K16.jpg]

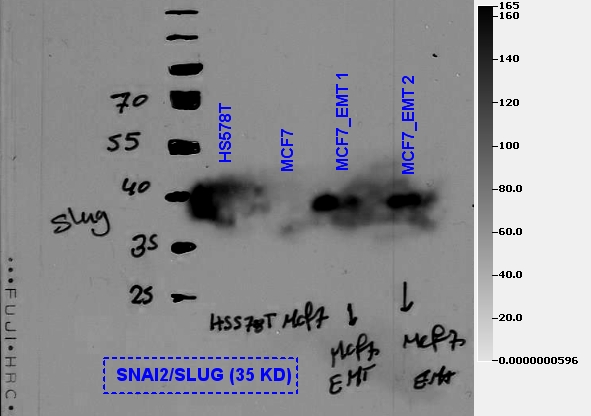

Supplement: Supplementary file 1 [file cancers-13-03869-s001.zip › cancers-1312672-supplmentary files/Original Images for Blots/EMT_SNAI2 (SLUG).jpg]

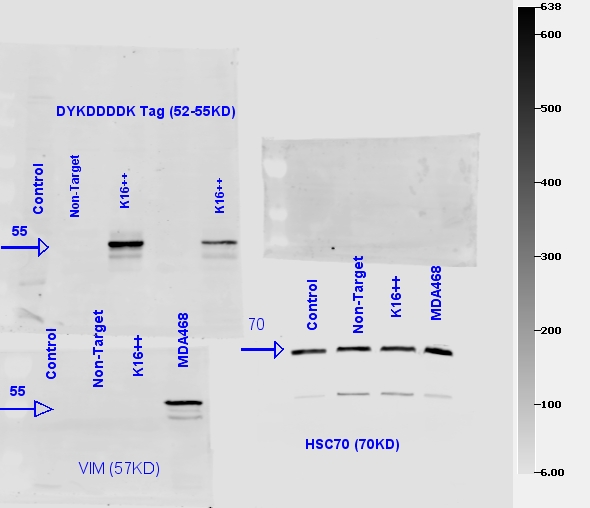

Supplement: Supplementary file 1 [file cancers-13-03869-s001.zip › cancers-1312672-supplmentary files/Original Images for Blots/K16++ (K16, VIM and HSc70).jpg]

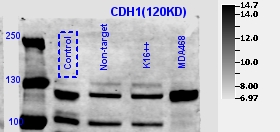

Supplement: Supplementary file 1 [file cancers-13-03869-s001.zip › cancers-1312672-supplmentary files/Original Images for Blots/K16++(CDH1).jpg]

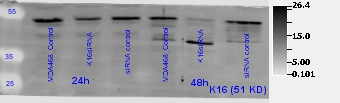

Supplement: Supplementary file 1 [file cancers-13-03869-s001.zip › cancers-1312672-supplmentary files/Original Images for Blots/siRNAK16 (K16).jpg]

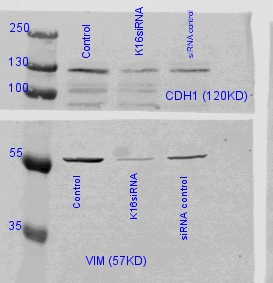

Supplement: Supplementary file 1 [file cancers-13-03869-s001.zip › cancers-1312672-supplmentary files/Original Images for Blots/siRNAK16(CDH1 and VIM).jpg]

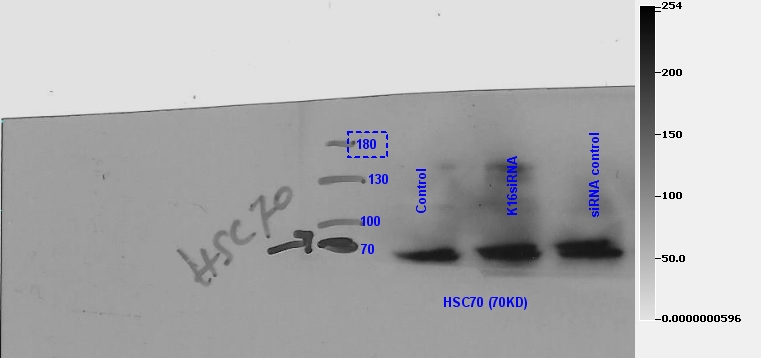

Supplement: Supplementary file 1 [file cancers-13-03869-s001.zip › cancers-1312672-supplmentary files/Original Images for Blots/siRNAK16(HSC70).jpg]
